# Non‐Invasive Assessment of Damping of Blood Flow Velocity Pulsatility in Cerebral Arteries With MRI

**DOI:** 10.1002/jmri.27989

**Published:** 2021-11-18

**Authors:** Tine Arts, Laurien P. Onkenhout, Raquel P. Amier, Rob van der Geest, Thijs van Harten, Jaap Kappelle, Sanne Kuipers, Matthijs J.P. van Osch, Ed T. van Bavel, Geert Jan Biessels, Jaco J.M. Zwanenburg, Heart‐Brain Connection Consortium

**Affiliations:** ^1^ Department of Radiology, UMCU Brain Center University Medical Center Utrecht Utrecht The Netherlands; ^2^ Department of Neurology, UMCU Brain Center University Medical Center Utrecht Utrecht The Netherlands; ^3^ Department of Cardiology Amsterdam Medical Center Location Vu Amsterdam The Netherlands; ^4^ C.J. Gorter Center for High Field MRI, Department of Radiology Leiden University Medical Center Leiden The Netherlands; ^5^ Amsterdam UMC, University of Amsterdam, Biomedical Engineering and Physics Amsterdam Cardiovascular Sciences Amsterdam The Netherlands

**Keywords:** damping, cerebral perforating arteries, velocity pulsatility, MRI

## Abstract

**Background:**

Damping of heartbeat‐induced pressure pulsations occurs in large arteries such as the aorta and extends to the small arteries and microcirculation. Since recently, 7 T MRI enables investigation of damping in the small cerebral arteries.

**Purpose:**

To investigate flow pulsatility damping between the first segment of the middle cerebral artery (M1) and the small perforating arteries using magnetic resonance imaging.

**Study Type:**

Retrospective.

**Subjects:**

Thirty‐eight participants (45% female) aged above 50 without history of heart failure, carotid occlusive disease, or cognitive impairment.

**Field Strength/Sequence:**

3 T gradient echo (GE) T1‐weighted images, spin‐echo fluid‐attenuated inversion recovery images, GE two‐dimensional (2D) phase‐contrast, and GE cine steady‐state free precession images were acquired. At 7 T, T1‐weighted images, GE quantitative‐flow, and GE 2D phase‐contrast images were acquired.

**Assessment:**

Velocity pulsatilities of the M1 and perforating arteries in the basal ganglia (BG) and semi‐oval center (CSO) were measured. We used the damping index between the M1 and perforating arteries as a damping indicator (velocity pulsatility_M1_/velocity pulsatility_CSO/BG_). Left ventricular stroke volume (LVSV), mean arterial pressure (MAP), pulse pressure (PP), and aortic pulse wave velocity (PWV) were correlated with velocity pulsatility in the M1 and in perforating arteries, and with the damping index of the CSO and BG.

**Statistical Tests:**

Correlations of LVSV, MAP, PP, and PWV with velocity pulsatility in the M1 and small perforating arteries, and correlations with the damping indices were evaluated with linear regression analyses.

**Results:**

PP and PWV were significantly positively correlated to M1 velocity pulsatility. PWV was significantly negatively correlated to CSO velocity pulsatility, and PP was unrelated to CSO velocity pulsatility (*P* = 0.28). PP and PWV were uncorrelated to BG velocity pulsatility (*P* = 0.25; *P* = 0.68). PWV and PP were significantly positively correlated with the CSO damping index.

**Data Conclusion:**

Our study demonstrated a dynamic damping of velocity pulsatility between the M1 and small cerebral perforating arteries in relation to proximal stress.

**Level of Evidence:**

4

**Technical Efficacy:**

Stage 1

## Introduction

Blood supply to the brain originates in the left ventricle of the heart and is conducted through the cardiovascular tree, propagated by pressure pulsations created by the cardiac contractions.^1^ The attenuation of these pressure pulsations as they travel towards the microcirculation of the brain is called damping. Damping of pressure pulsations not only occurs in the large arteries such as the aorta, carotid arteries, and the circle of Willis (CoW), but also beyond the CoW, up to the small arteries and the microcirculation.^2^, ^3^, ^4^ As such, damping prevents excessive pulsatile energy from reaching the microcirculation and averts potential cerebral damage.^5^, ^6^

Methods for obtaining proximal measures of pressure and arterial stiffness, such as mean arterial pressure (MAP), aortic pulse wave velocity (PWV), and pulse pressure (PP) are commonly available, as well as tools to measure pressure in more distal vessels such as the carotid arteries and arteries of the CoW. For example, 3 T magnetic resonance imaging (MRI) allows aortic PWV measurements can assess arterial stiffness, and cardiac 3 T MRI is used to assess left ventricular stroke volume (LVSV). However, technology did not enable non‐invasive pressure measurements in the smaller cerebral arteries, preventing investigation of damping beyond the CoW, i.e., cerebral damping, and its relation with proximal measures of pressure and arterial stiffness in humans. Better insight into cerebral damping of pressure pulsations would not only advance our understanding of normal physiology, but could also shed light on the relationship between vascular disease or hypertension and the development of brain damage.^6^

With 7 T MRI phase contrast (PC) MRI, it has become possible to measure blood velocity pulsatility in the small cerebral perforating arteries.^7^, ^8^ This allows the assessment of blood flow velocity pulsatility in patients with small vessel disease.^9^ Although previous studies investigated changes of velocity pulsatility within the brain with 7 T MRI, i.e., cerebral damping, relations of damping with proximal stress have not been previously investigated.^10^ Therefore, in this study, our aim was to assess the presence of damping (which is expressed as a damping index) between the first segment of the middle cerebral artery (M1) and the perforating arteries, and, secondly, investigate relations between proximal measures of pressure and arterial stiffness, and the measured damping index.

## Materials and Methods

### Study Population and Study Visits

The data used in this study originated from the Heart‐Brain Connection study, which was approved by the METCs and local boards of the participating institutions.^11^ The Heart Brain Connection study was conducted in accordance with the declaration of Helsinki and the Medical Research Involving Human Subjects Act (WMO). All included participants provided written informed consent, and were recruited through advertising leaflets in the hospital or were spouses of patients participating in the same program. The participants included in our study originated from the reference group of the Heart Brain Connection study^11^ and were aged >50 years. Contrary to the non‐reference group, these participants did not have a history of heart failure, carotid occlusive disease, or cognitive impairment. All participants underwent clinical assessment of sociodemographic factors and cardiovascular risk factors, extensive neuropsychological testing, an assessment of daily functioning, psychiatric measures, and blood pressure measures. Furthermore, a venous blood sample was drawn to assess biomarkers. In addition, cardiac (3 T MRI) and brain MRI (3 T and 7 T MRI) images were acquired. The obtained blood pressure measures and MRI images were used in the current study.

### MRI Scanners

3 T and 7 T MRI data were acquired at two institutions. 3 T MRI was used for obtaining proximal measures of pressure and arterial stiffness, together with conventional brain scanning to capture brain anatomy and the white matter lesions. The 7 T MRI scanner was used for assessing the distal vessels, in particular, the perforating arteries that cannot be assessed with sufficient sensitivity at 3 T. Details of the 3 T and 7 T MRI acquisitions are provided below.

#### 3 T MRI

At both institutions, 3 T MRI data were acquired on a Philips Ingenia MRI scanner. One institution used an 8‐channel receive head coil for the brain MRI scans, and the other a 32‐channel head coil (Philips, Best, The Netherlands). At 3 T MRI a T1‐weighted image (T1WI) (1.0 mm × 1.0 mm × 1.0 mm resolution; echo time/repetition time (TE/TR) = 4.5/7.9 msec; flip angle = 8°; sense factor = 2; field of view (FOV) = 256 mm × 256 mm × 192 mm) and a spin echo fluid‐attenuated inversion recovery (FLAIR) (1.0 mm × 1.0 mm × 1.0 mm resolution; TE/TR = 275/4800 msec; flip angle = 90°; inversion time = 1650 msec; sense factor = 2; FOV = 224 mm × 224 mm × 160 mm) of the brain was acquired. These images enabled delineations of white matter and infarcts, respectively, which were automatically performed using the Quantib Brain Segmentation Tool (Quantib. B.V., Rotterdam, The Netherlands). To ensure complete inclusion of infarction zones, the infarct masks were dilated with a 3 mm × 3 mm kernel.

In addition, a T1WI and 2D PC data were recorded for PWV measurements (scan parameters are provided below) as well as a cine steady‐state free precession (SSFP) sequence (scan parameters are provided below) for LVSV measurements.

#### 7 T MRI

At both institutions, 7 T MRI data were acquired on a Philips Achieva MRI system with a 32‐channel receive head coil (Nova Medical, Wilmington, MA). A short T1WI was acquired for alignment between 3 T and 7 T MRI brain images (1.0 mm × 1.0 mm × 1.0 mm resolution, TR/TE = 4/1.9 msec, flip angle = 7°, sense factor = 1, FOV = 300 mm × 300 mm × 190 mm). Further, intracranial pulsatility measurements of the M1 and the small perforating arteries were performed (scan parameters are provided below). These measurements were acquired approximately 3 months after.

### Data Acquisition and Processing: Intracranial Pulsatility Measures

#### FIRST SEGMENT OF THE MIDDLE CEREBRAL ARTERY (M1)

Velocity (cm/second) and pulsatility measurements at the first segment of both left and right middle cerebral artery (MCA) (M1) were acquired on 7 T MRI using a 2D quantitative flow (Qflow) gradient echo (GE) sequence (0.5 mm × 0.5 mm resolution; slice thickness = 3 mm; TR/TE = 18.5/7.7 msec; flip angle = 60°; Venc = 120 cm/second; sense factor = 1; FOV = 250 mm × 250 mm; scan duration = 114 seconds for a heart rate of 60 bpm). A peripheral pulse unit was used for retrospective gating and cushions were placed beside the subject's head to minimize head movement during scanning. Contouring of the M1 was done semi‐automatically using MeVisLab (v3.1.1, VS2017, MeVis Medical Solutions AG, Bremen, Germany) and M1 velocity pulsatility was calculated using the flow velocity, where flow velocity was defined as the average over the contoured lumen. Blood flow velocity pulsatility was derived from the velocity curve using the following formula:

(1) $$\text{Pulsatility}=\frac{V_{\max}-V_{\min}}{V_{\text{mean}}},$$

where *V*_max_, *V*_min_, and *V*_mean_ are the maximum, minimum, and mean flow velocity respectively. Pulsatility values of left and right M1 were averaged. Participants with flow voids accompanied by visible noise in the velocity trace were excluded. To this end, 2D‐Qflow scans were qualitatively evaluated by two observers (TA, 3 years of experience; JJMZ, 15 years of experience). If both observers visually observed flow voids in the phase or magnitude image as well as visible noise in the velocity trace, a participant was excluded.

#### CEREBRAL PERFORATING ARTERY FLOW VELOCITY AND PULSATILITY

2D PC GE acquisitions aimed at small perforating arteries in the semi‐oval center (CSO) and the basal ganglia (BG) were performed on 7 T MRI using a previously published sequence (0.3 mm × 0.3 mm resolution; slice thickness = 2 mm; reconstructed resolution = 0.18 mm × 0.18 mm; turbo field echo factor = 2; phase encoding in anterior–posterior direction) (CSO: TE/TR = 29/16 msec; flip angle = 50–90° using tilted optimized nonsaturating excitation (TONE)^7^; Venc = 4 cm/second; Sense factor = 1.5; FOV = 250 mm × 250 mm) (BG: TE/TR = 28/15 msec; flip angle = 60°; Venc = 20 cm/second; sense factor = 1; FOV = 250 mm × 160 mm).^7^, ^8^ Similar to the Qflow acquisition, a peripheral pulse oximeter was used for retrospective gating and cushions were placed beside the subject's head to minimize head movement during scanning. Before analysis, 2D PC scans were visually checked for sufficient quality by one rater (TA; 3 years of experience) at two time points, 2 weeks apart. At both time points images were classified with either “sufficient quality” or “insufficient quality” based on visual evaluation of motion artifacts and planning location. If either motion artifacts or incorrect planning location was expected to lead to unreliable results, the scan was classified with “insufficient quality.” Scans classified with “insufficient quality” at both time points were excluded from analysis.

Perforating artery flow analysis of the 7 T MRI 2D PC scans was performed in specified regions of interest of the CSO and BG. To enable the establishment of these regions of interest in 7 T space, 3 T delineations of the white matter and infarcts were registered to 7 T space. Registration of the delineations to 7 T PC space was achieved using the transformation of the 3 T T1WI to the 7 T T1WI with the FMRIB Software Library (FSL version 6.0.1, Oxford, UK). With the delineations in 7 T space, a white matter mask was constructed by excluding the infarct delineations from the white matter delineations. Concerning the CSO scan, perforating artery flow was assessed in the white matter using the white matter mask. Only the central white matter (beyond 16 mm of the rim of the brain on the PC brain slice) was included because the poor gray/white matter contrast in these regions on the PC image makes it difficult to recognize a mismatch between the white matter mask and the underlying anatomy, which could result from subject motion between the acquisition of the anatomical T1WI and the 2D PC acquisition. Concerning the BG scan, perforating artery flow was assessed in the region between the insula and the ventricles. This region was manually delineated and excluded the automatically delineated infarcts registered to 7 T space. Cerebral perforating arteries in the CSO and BG were detected automatically as previously published using Matlab (R2015b, The MathWorks, Natick, MA, USA),^7^, ^8^ excluding CSO perforating arteries located in ghosting artifact regions^12^ and excluding perforating arteries in the BG oriented non‐perpendicularly to the scanning plane. In addition, apparent perforating arteries located within a 1.2‐mm radius from each other were excluded, as in our experience these are mostly “false detections” located on larger and non‐perpendicular vessels.^12^

For each detected perforating artery, an average blood‐flow velocity was obtained. The mean blood‐flow velocity curve per subject was determined by averaging over all perforating arteries. To calculate the velocity pulsatility, the perforating artery's velocity curves were first normalized by division by the mean and then averaged. The velocity pulsatility was subsequently calculated with Eq. 1 (note: due to the normalization procedure, *V*_mean_ equals 1.0). Perforating artery count within the regions of interest was expressed as a density (number of perforating arteries/cm^2^ mask, *N*_density_).

#### DAMPING INDEX

The damping index was defined using the following formula^13^:

$$\text{Damping index}=\frac{{\text{Velocity pulsatility}}_{M1}}{\text{Velocity}\;{\text{pulsatility}}_{\mathrm{CSO}/\mathrm{BG}}}.$$

This damping index reflects the amount of damping between the M1 and cerebral perforating arteries using one measure (a damping index >1.0 represents damping, while a damping index <1.0 reflects an increase in velocity pulsatility rather than damping).

### Data Acquisition and Processing: Proximal Measures of Pressure and Arterial Stiffness

#### LEFT VENTRICULAR STROKE VOLUME

LVSV (mL) was used as a pressure measure as the pressure measure PP is highly associated with LVSV; the PP wave results from systolic ejection of blood from the left ventricle which is followed and impacts diastolic arterial dissipation of the LVSV.^14^, ^15^ LVSV was measured with 3 T MRI using a breath‐hold short‐axis multi‐slice cine balanced GE SSFP sequence (1.25 mm × 1.25 mm resolution; TR/TE = 3.8/1.9 msec; flip angle = 45°; 40 heart phases; breath‐hold; number of slices dependent on LV (range 12–16 slices); 67 phase percentage; slice thickness = 8 mm; sense factor = 2; FOV = 500 mm × 280 mm).^11^ Analysis was done using MASS research software (v2018‐EXP, Leiden, The Netherlands) and consisted of the following steps: 1) using cine mode visualization of the images, the end‐diastolic and end‐systolic phases were determined. End‐diastolic was defined as the phase of maximum left ventricular cavity and end‐systolic as the phase of minimum left ventricular cavity volume; 2) using the semi‐automated contour detection of MASS, left ventricular endocardial contours were generated for the imaging slices from apex to base in the end‐diastolic and end‐systolic phases; 3) left ventricular end‐diastolic volume and left ventricular end‐systolic volume were derived; 4) LVSV = (left ventricular end‐diastolic volume − left ventricular end‐systolic volume) was calculated.

#### BLOOD PRESSURE

To determine MAP (mmHg) and PP (mmHg), systolic and diastolic blood pressure (in mmHg) were acquired in a standardized way. Each visit the patient was asked to sit for 5 minutes, after which the blood pressure was measured on each arm and repeated at least 1 minute later. Systolic and diastolic blood pressure were acquired by averaging the second measurements of the two study visits. The MAP was calculated with the following formula:

$$\mathrm{MAP}=\left(2*\text{diastolic blood pressure}+\text{systolic blood pressure}\right)/3.$$

PP was calculated by taking the difference between mean systolic and diastolic blood pressure.

#### AORTIC PULSE WAVE VELOCITY

The aortic arch was imaged on 3 T MRI using a multi‐slice 3D T1‐weighted GE acquisition in oblique sagittal orientation (reconstructed into 15 slices with a voxel size of 1.76 mm × 1.76 mm, slice thickness = 5 mm, flip angle = 15°, FOV = 400 mm × 300 mm, TR/TE = 4.9/2.4 msec, sense factor = 1). Additionally, a 2D phase GE contrast velocity mapping sequence was acquired intersecting both ascending and descending aorta at the level of the pulmonary artery bifurcation (2.5 mm × 2.5 mm resolution; slice thickness = 8 mm; TR/TE = 4.7/2.8 msec; flip angle = 10°; Venc = 150 cm/second; sense factor = 1; FOV = 320 mm × 320 mm; scan duration = 107 seconds for a heart rate of 60 bpm; temporal resolution is 5 msec; the number of acquired cardiac phases was 186 for a heart rate of 62 bpm, and scaled proportionally to the cardiac cycle length of each individual). Therefore, the number of acquired cardiac phases depended on the heart rate.^11^ Quantification of PWV from these acquisitions was performed using a fully automated method as previously described.^16^ In brief, the aortic arch was segmented, from which the 3D aortic center lumen line was derived. The two intersection points of the centerline with the velocity mapping slice were used as seed points for automated segmentation of the ascending and descending aorta and time velocity curves were derived for both ascending and descending aorta. The pulse wave transit time from ascending to descending aorta was derived using the half‐max method and the traveled distance from ascending to descending aorta was measured along the computed centerline. PWV was subsequently derived according to the following formula:

$$\mathrm{PWV}=\frac{l}{∆t},$$

where *l* is the distance along the centerline between the ascending and descending aorta cross‐section and ∆*t* is the difference in pulse‐wave arrival time. Aortic PWV is measured in m/second.^16^

### Statistical Analysis

The relations between proximal measures of pressure and arterial stiffness, velocity pulsatility in the M1 and in the small cerebral perforating arteries, as well as relations with the damping indices were evaluated using correlation coefficients, derived from linear regression analyses. To facilitate comparison between pulsatility, pressure, and arterial stiffness measures along the cardiovascular tree and to provide confidence intervals, all data were presented as standardized beta (B [95% CI]). All data analyses were performed in IBM SPSS statistics (IBM Corp., v22.0, NY, USA). A *P*‐value ≤0.05 was considered statistically significant.

## Results

In Fig. S1 of the Supplemental Material, an overview of the 3 T and 7 T MRI scans used for this research are shown.

2D PC scan quality was sufficient in 33 of 38 subjects, of which 28 and 26 subjects had sufficient scan quality in the CSO and BG, respectively. In the CSO, 10 subjects were excluded due to excessive subject motion. In the BG, nine and three subjects were excluded due to motion and erroneous planning.

**FIGURE 1:**
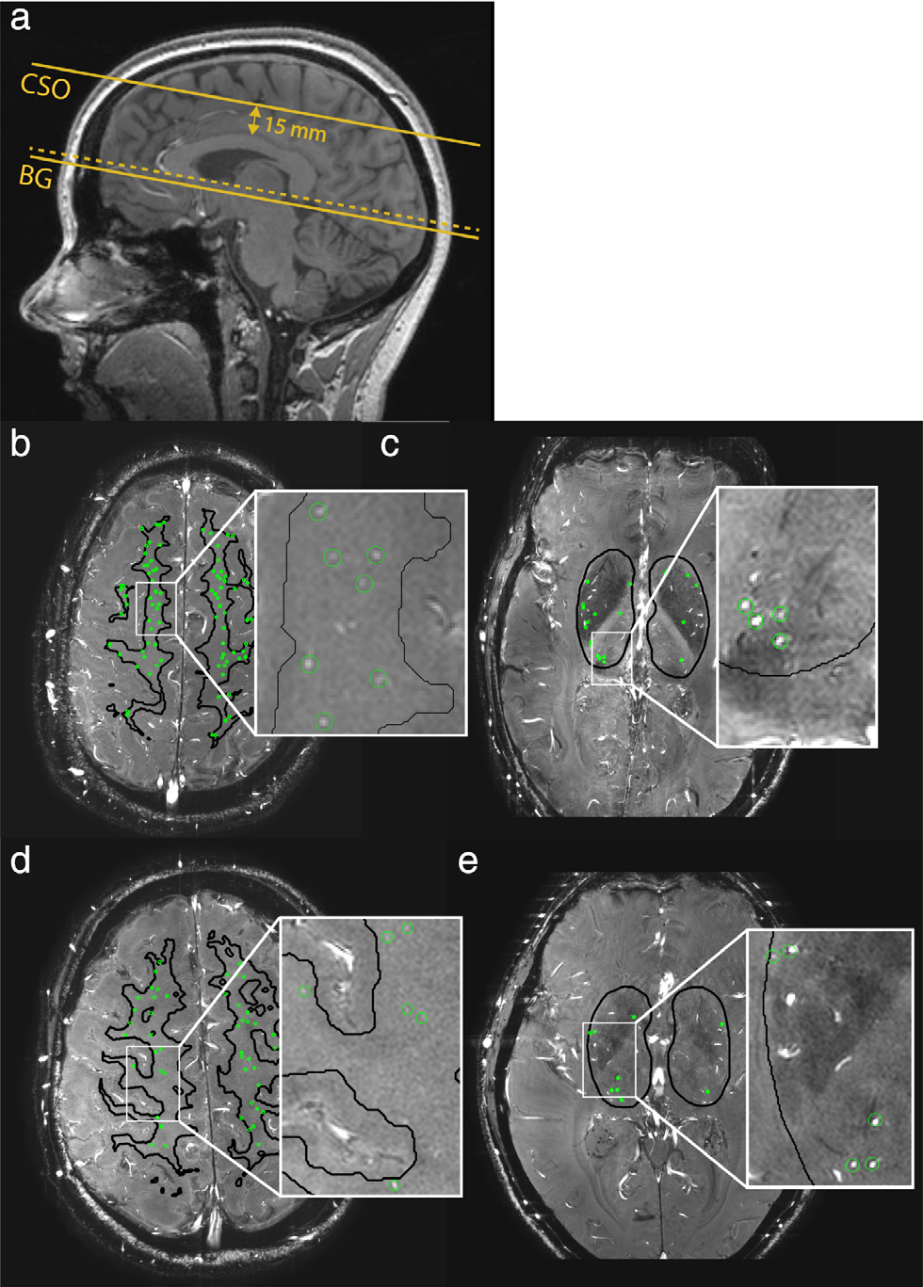
Slice planning of the phase contrast (PC) images and detected perforating arteries. (**a**) Location of the 2D PC slice in the semi‐oval center (CSO), located 15 mm above the corpus callosum, and in the basal ganglia (BG) located at the level of the anterior commissure. Slice angulation is associated with the bottom of the corpus callosum (dotted line). (**b** and **d**) Two subjects are shown, and cerebral perforating arteries detected in the CSO are circled in green. (**c** and **e**) Two subjects are shown, and cerebral perforating arteries detected in the BG are circled in green.

Included participants were aged 65 ± 8 years (mean ± SD), and 55% were men. None had diabetes, 26% had hypertension, and 8% had a history of cardiovascular disease (i.e., stroke, TIA, or ischemic heart disease). Cardiac measures, as well as blood pressure and vascular measures, are presented in Table 1. Figure 1 shows the slice planning of the CSO and BG 2D PC scan, as well as the small vessels detected in these two regions.

**TABLE 1:** Cardiac Measures, Blood Pressure, and Vascular Measures

Demographics	
Age (years)	65 ± 8
Sex (male)	18 (55)
Vascular risk factors	
Hypertension	9 (26)
Hypercholesterolemia	10 (29)
Diabetes mellitus	0 (0)
Current smoking	2 (6)
History of reported TIA	1 (3)
History of reported ischemic stroke	0 (0)
Cardiac	
Left ventricular stroke volume (mL)	91.3 ± 20
Blood pressure	
Systolic blood pressure (mmHg)	135 ± 19
Diastolic blood pressure (mmHg)	81 ± 11
MAP (mmHg)	99.8 ± 13.3
Pulse pressure (mmHg)	54.6 ± 14.3
Aorta	
Pulse wave velocity (m/second)	8.2 ± 2.9
Middle cerebral artery (M1) (*N* = 32)	
Velocity (cm/second)	35 ± 7.5
Pulsatility	0.76 ± 0.17
Cerebral perforating arteries	
Basal ganglia (BG; *N* = 26)	
*N*_density_ (# perforating arteries/cm^2^)	0.66 ± 0.22
Velocity (cm/second)	3.8 ± 0.80
Pulsatility	0.40 ± 0.14
Semi‐oval center (CSO; *N* = 28)	
*N*_density_ (# perforating arteries/cm^2^)	2.6 ± 1.0
Velocity (cm/second)	0.7 ± 0.12
Pulsatility	0.42 ± 0.13
Damping index	
Between M1 and BG (*N* = 26)	2.1 ± 0.81
Between M1 and CSO (*N* = 28)	2.0 ± 0.93

Data presented as group mean ± SD or *N* (%). *N* = 33 unless otherwise indicated.

MAP = mean arterial pressure.

**FIGURE 2:**
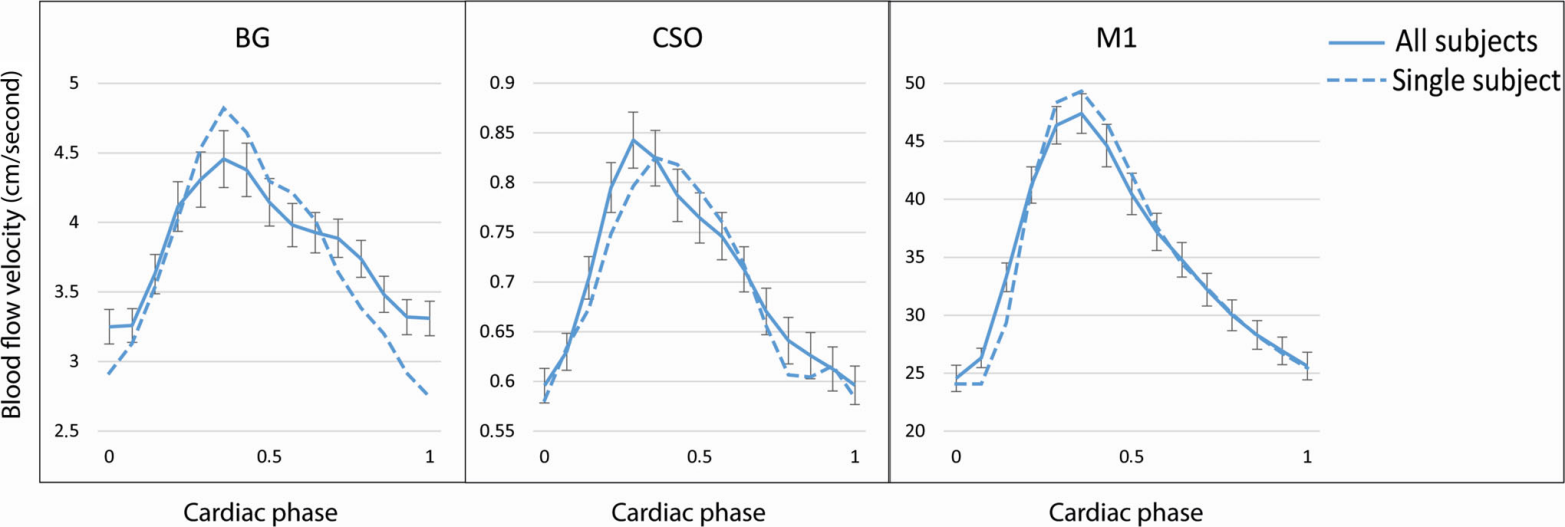
Blood flow velocity curves of the basal ganglia (BG), semi‐oval center (CSO), and middle cerebral artery (M1). Mean velocity curves over all subjects are represented by the solid lines, velocity curves of a single subject are represented by the dotted lines. Error bars show ±standard error of the mean. Curves were interpolated to the maximum number of cardiac phases present, i.e., 15.

**FIGURE 3:**
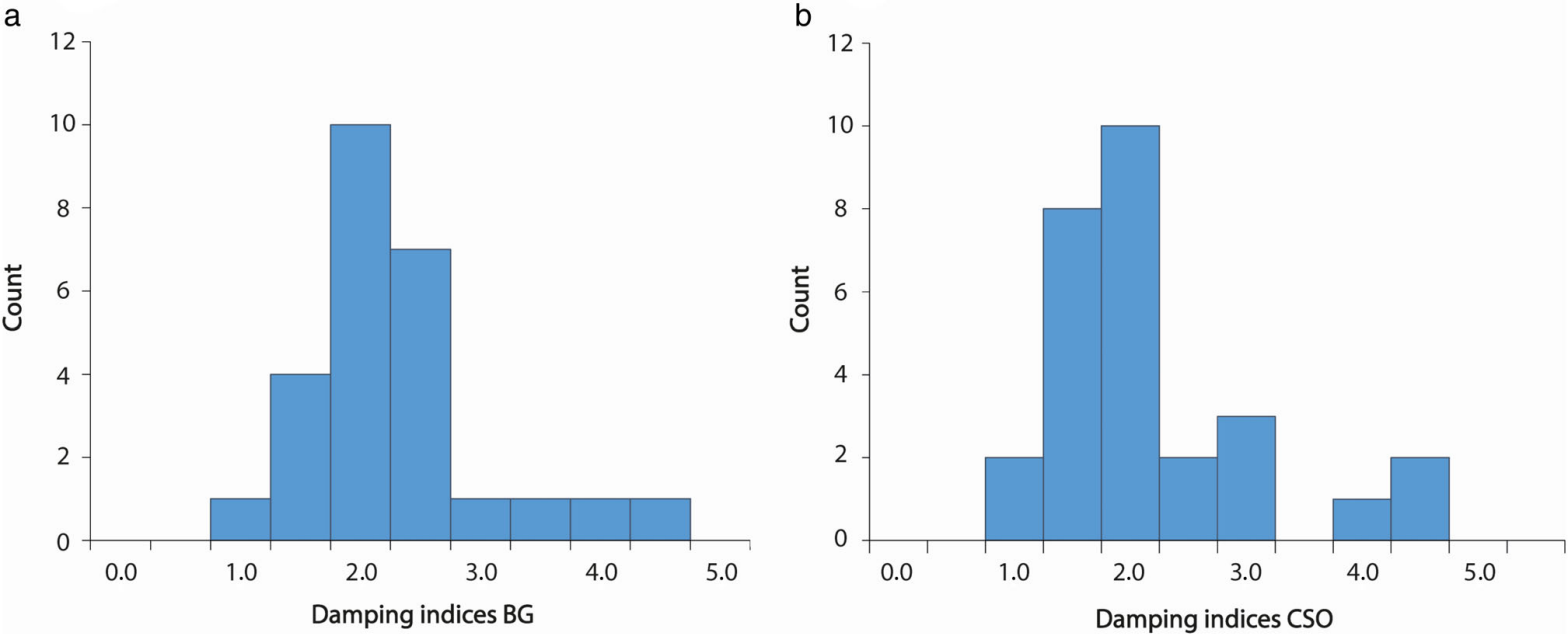
Bar graphs showing the distribution of the damping indices of all subjects in the basal ganglia (BG; *N* = 26) (**a**) and the semi‐oval center (CSO; *N* = 28) (**b**).

Velocity pulsatility in the M1 was significantly higher compared to the velocity pulsatility in the perforating arteries of the CSO and BG. This resulted in the damping index between the M1 and CSO (i.e., CSO damping index) and between the M1 and BG (i.e., BG damping index) to be larger than 1.0 in all but one subject, (in whom the measured CSO damping index was 0.94). The CSO and BG damping indices were similar (*P* = 0.74). These results are shown in Table 1. In Fig. 2, the mean velocity curves of all subjects are shown in the BG, CSO, and M1, as well as velocity curves of a single subject. In Fig. 3, bar graphs show the distribution of the damping indices in the CSO and BG of all subjects.

PP and aortic PWV were significantly positively correlated to M1 velocity pulsatility (0.47 [0.06, 0.87] and 0.49 [0.08, 0.90]). By contrast, aortic PWV was negatively correlated to CSO velocity pulsatility (−0.47 [−0.83, −0.10]) and PP was not correlated to CSO velocity pulsatility (−0.21 [−0.60, 0.18], *P* = 0.28). PP and aortic PWV were also not correlated to BG velocity pulsatility (0.23 [−0.18, 0.65], *P* = 0.25 and 0.09 [−0.34, 0.52], *P* = 0.68, respectively). A significant correlation was found between aortic PWV and the CSO damping index (0.71 [0.40, 1.02]), and the same was seen for PP and the CSO damping index (0.49 [−0.10, 0.88]). Results of the correlations between proximal measures of pressure and arterial stiffness, velocity pulsatility in the M1 and in the small cerebral perforating arteries are shown in Table 2. For illustrative purposes, Fig. S2 in the Supplemental Material shows relations of aortic PWV with BG, CSO and M1 velocity pulsatility, PP with BG, CSO and M1 velocity pulsatility, aortic PWV with BG and CSO damping indices, and a mean‐split scatterplot of CSO pulsatility and M1 pulsatility and the association with aortic PWV.

**TABLE 2:** Correlations Between Proximal Measures of Perfusion Pressure and Arterial Stiffness, Flow Pulsatility in the Middle Cerebral Artery and in the Small Cerebral Perforating Arteries

	M1 Pulsatility (*N* = 32)	*P*	BG Pulsatility (*N* = 26)	*P*	Damping Index M1/BG (*N* = 26)	*P*	CSO Pulsatility (*N* = 28)	*P*	Damping Index M1/CSO (*N* = 28)	*P*
LVSV (mL)	0.07 [−0.40, 0.55]	0.75	0.06 [−0.37, 0.49]	0.78	−0.26 [−0.71, 0.19]	0.24	−0.18 [−0.59, 0.24]	0.39	0.01 [−0.45, 0.46]	0.98
MAP (mmHg)	−0.10 [−0.55, 0.35]	0.65	−0.23 [−0.61, 0.16]	0.23	0.17 [−0.31, 0.64]	0.47	−0.18 [−0.55, 0.20]	0.34	0.07 [−0.37, 0.51]	0.76
PP (mmHg)	0.47 [0.06, 0.87]	0.03*	0.23 [−0.18, 0.65]	0.25	0.32 [−0.14, 0.77]	0.16	−0.21 [−0.60, 0.18]	0.28	0.49 [0.10, 0.88]	0.02*
Aortic PWV (m/second)	0.49 [0.08, 0.90]	0.02*	0.09 [−0.34, 0.52]	0.68	0.20 [−0.25, 0.66]	0.37	−0.47 [−0.83, −0.10]	0.01*	0.71 [0.40, 1.02]	0.00*
M1 pulsatility	—	—	0.30 [−0.23, 0.82]	0.25	—	—	0.10 [−0.41, 0.61]	0.68	—	—

Data are presented as standardized regression coefficients (B) with 95% CI. Statistical results were obtained using linear regression analysis.

LVSV = left ventricular stroke volume; MAP = mean arterial pressure; PP = pulse pressure; PWV = pulse wave velocity; M1 = first segment of the middle cerebral artery; BG = basal ganglia; CSO = semi‐oval center.

* A *P*‐value ≤0.05 was considered significant and marked.

## Discussion

This study showed the presence of damping between the M1 and perforating arteries in the BG and CSO, which was evident from the damping indices (>1.0). Variable damping behavior was suggested, given the positive correlations between proximal measures of pressure and arterial stiffness with the pulsatility in the M1, and lacking or negative correlations with pulsatility in the more distal perforating arteries in the CSO and BG. This was furthermore implied by the positive correlations between proximal measures of pressure and arterial stiffness with the damping index between M1 and CSO. Thus, these findings not only indicated the presence of damping between the M1 and the perforating arteries of the CSO and BG, but also suggested that the damping was variable between subjects in a way that distal damping was increased when proximal pressure and arterial stiffness increased.

The values found in our cohort for the velocity pulsatility in the M1 matched well with those found in the literature, which range between 0.56 and 0.97 for controls without stroke or white matter hyperintensities of various ages.^8^, ^10^, ^17^, ^18^, ^19^, ^20^, ^21^ Velocity pulsatility in the cerebral perforating arteries has been less studied. The values reported here agree with those reported previously.^8^, ^9^ Schnerr et al reported a PI of 0.46 in the lenticulostriate arteries for younger subjects (mean age 25 years) and 0.69 for older subjects (mean age 75 years), which is higher than our values.^10^ This may partly be explained by the fact that Schnerr et al analyzed only the relatively large perforating arteries close to the MCA with a mean perforating artery diameter of 1.7 mm. This is also reflected in the lower damping index found by the authors between those vascular segments (1.31 ± 0.30 vs. 2.1 ± 0.81 in this study). The proximal measures of pressure and arterial stiffness assessed in this study were similar to those reported in the literature.^19^, ^22^, ^23^, ^24^, ^25^ Slight differences were likely due to the fact that we included participants with variable levels of risk factors and vascular events, which assured us of the required variation in the measures of proximal pressure and arterial stiffness. In case of too little variation in the measurements, observed variability would only be noise and not contain information from which relations can be inferred.

The correlations found in this study between proximal measures and pulsatility and damping indices in the CSO and BG indicated variable damping behavior, in which damping appeared to increase with increased proximal “stress,” thus mitigating the effect on the pulsatility in the microcirculation. The suggestion of the existence of mechanisms that mitigate pressures traveling towards the microcirculation is not new, though techniques to measure flow pulsations in the small cerebral vessels were previously lacking.^26^ Nevertheless, we can only speculate about the potential mechanisms underlying the negative correlation of PWV with CSO velocity pulsatility. Increased PWV indicates increased aortic stiffness, but it is also suggested to indicate arterial stiffness in other cerebral vascular beds.^27^ Arterial stiffness affects propagation and reflection characteristics of pressure waves of the arterial tree.^6^ Given these changes in reflection, we could speculate that, eg, in the pial arteries which feed the CSO and not the BG, cancelation of forward and backward traveling waves occurs, decreasing CSO pulsatility. However, Mitchell et al suggest a more proximal role for wave reflections.^5^ Their research states that an increase in aortic stiffness as compared with the carotid arteries results in a reduction of wave reflections at the interface of the aorta and carotid arteries, which would increase pulsatile energy towards the microcirculation, contrary to our findings.

In this study, the presence of positive correlations of PP and PWV with M1 and the lack of correlations of PP and PWV with BG pulsatility suggested damping. Though, counterintuitively, correlations between PP and PWV with the BG damping index were not found. This may be due to the fact that the pulsatility measures are relatively noisy, which is known from earlier studies.^7^, ^12^ As is known from error propagation in parameter estimation, the division of two (noisy) pulsatility measures, i.e., the damping index, increases the level of noise.^28^ It may thus be that a weak correlation with the BG damping index exists, but was lost in the noise. Also, correlations of PP and PWV with M1 pulsatility showed moderate (<0.5) correlations which suggest some individual variability in how M1 pulsatility changes in relation to changes in PP and PWV. This intersubject variability may also have caused that correlations of PP and PWV with the BG damping index were too weak to be measured. Nevertheless, our results suggested “compensatory” damping between the M1 and CSO perforating arteries to be stronger compared to damping between the M1 and BG perforating arteries. This difference may be due to the fact that the small perforating arteries of the CSO are several branches further away from the M1 compared to the small perforating arteries of the BG.^29^ In addition, pathology and pathological degeneration of the perforating arteries in the BG and CSO are different, which is reflected by the fact that arteriolar and tissue damage in the BG advances that in the CSO, and is more severe.^29^ Finally, it is known that blood supply to the CSO, contrary to that of the BG, occurs via the pial arteries originating from the MCA,^30^, ^31^ and that pial arteries attenuate pressure and flow pulsatility prior to their arrival in downstream tissues.^32^

Research into the damping of flow pulsations traveling from the heart towards the cerebral vasculature is not new. Damping of flow pulsations is known to be associated with arterial compliance, i.e., the ability of elastic vessels to stretch and recoil and, with that, absorb pulsatile energy and protect the cerebral vasculature.^5^

It is believed that the aorta, with its high compliance, plays a primary role in the absorption of pulsatile energy, Further, wave reflections in the relatively stiff muscular arteries as well as the geometry of the CoW, which makes it serve as a pressure damper, play a role in pulsatile energy absorption.^19^, ^33^ According to standard physiology textbooks, compliance of the cerebral small arteries and arterioles, located just proximal to the capillaries, is also important for pressure dampening.^2^, ^3^, ^4^ These vessels are lined with a smooth muscle cell layer enabling active alterations in compliance by changes in tone or wall structure.^34^, ^35^ While research into cerebral damping of flow pulsations is not new, research into damping of flow pulsations distal from the CoW is scarce. Earlier research into cerebral pulsatility and damping mainly focused on the larger arteries such as the carotid arteries, basilar artery, and the anterior, posterior, and MCA.^4^, ^36^ Recently, velocity pulsatility of smaller cerebral arteries was performed with 4D flow MRI and by averaging over many planes and vessels.^37^ This enabled the assessment of distal cerebral arteries originating from cerebral cortical arteries. However, this method was unable to assess vessels with diameters smaller than approximately 1 mm, and an assessment of regional vessel flow is difficult and time‐consuming. Nonetheless, this study did find a cerebral arterial pulsatility increase as a function of age. A similar finding was observed in the large perforating arteries assessed by Schnerr et al,^10^ revealing an increase in pulsatility and a decrease in damping factor with age. These studies are in agreement with the belief that pulsatile energy in the small cerebral arteries increases with age and contributes to the development of small vessel disease and manifestation such as white matter hyperintensities and cognitive decline. Research into brain pulsatility with near‐infrared spectroscopy (NIRS) also showed a significantly higher pulsatility in older compared to younger adults.^38^ In addition, NIRS brain pulsatility in relation to cortical thickness (NIRS) implied autoregulation mechanisms to damp and regulate pulsatility distal from the internal carotids.^38^ With this work, we extended the current literature on pulsatility damping to damping beyond the CoW up to the level of the perforating arteries in the white matter of the CSO. In addition, we have shown that with 2D PC imaging at 7 T MRI damping of the cerebral small vessels can be investigated. This paved the way for future research of damping of cerebral pressure pulsations in other cohorts, such as young healthy subjects or subjects with cardiovascular disease due to a potentially decreased ability to dampen pressure pulsations in the cardiovascular tree.

### Limitations

First, the PC sequence is relatively sensitive to motion during scanning. This can result in severe motion artifacts and give unreliable velocity and pulsatility measurements.^39^ This led to a considerable number of subjects to be excluded from small perforating artery analysis for the CSO level and BG level. This high exclusion rate is also known from earlier studies^7^, ^9^ and so far no quality assessment method has been developed to objectively exclude PC images from analysis. We therefore relied on an experienced observer to decide whether the quality was sufficient. Although data of the elderly and diseased is more likely to be excluded due to severe motion artifacts, we believe that heterogeneity of our cohort, i.e., with multiple and variable levels of risk factors and vascular events, makes exclusion bias unlikely. A second limitation was that the perforating artery detection method inherently has a detection threshold, making perforating arteries with flow velocities below this threshold undetectable. Such floor‐effects may in particular affect the CSO measures due to smaller perforating arteries and thus lower signal‐to‐noise compared to the measures in the BG.^7^, ^9^ Third, for the M1 2D‐Qflow sequence, a long echo time resulting in flow voids resulted in an exclusion of Qflow scans. Therefore, seven subjects had only either left or right M1 measurements, with no possibility of averaging both measurements. However, all study participants were healthy controls, and we, therefore, expected no large differences between left and right M1 flow pulsatility. A final limitation relates to the fact that the perforating artery pulsatility measure was relatively noisy. Therefore, the absence of a correlation between pressure measures and the somewhat noisy perforating artery pulsatility does not necessarily mean that an effect between these measures does not exist. It is therefore important that in the future, the 2D PC perforating artery pulsatility measures are supplemented with other pulsatility measurements, such as NIRS, and that more prospective studies be performed.

## Conclusions

The results presented in this paper showed the presence of damping between the M1 and small cerebral perforating arteries of the CSO and BG as well as variable damping behavior in relation to changes in proximal pressure and arterial stiffness.

## Conflict of Interest

The authors declare there are no conflicts of interest.

## Supporting information


**Fig S1** Overview of the MRI scans used for the discussed research, on 3 T and 7 T MRI. For the scans pertaining to the middle cerebral artery, the arrows indicate the M1.
**Fig S2**. Scatterplots showing relations of aortic pulse wave velocity with BG, CSO and M1 velocity pulsatility, pulse pressure with BG, CSO and M1 velocity pulsatility, pulse wave velocity with BG and CSO damping indices, and a mean‐split scatterplot of CSO pulsatility and M1 pulsatility, and the association with pulse wave velocity.

## Data Availability

The anonymized patient data that support the findings in this study are available from the corresponding author upon reasonable request.
