# Supplementary material for: Non‐Invasive Assessment of Damping of Blood Flow Velocity Pulsatility in Cerebral Arteries With MRI
Source: J Magn Reson Imaging. 2021 Nov 18;55(6):1785–94. doi: 10.1002/jmri.27989 (PMC9298760; doi:10.1002/jmri.27989)
Supplement: Supplementary file 1 — Fig S1 Overview of the MRI scans used for the discussed research, on 3 T and 7 T MRI. For the scans pertaining to the middle cerebral artery, the arrows indicate the M1. Fig S2. Scatterplots showing relations of aortic pulse wave velocity with BG, CSO and M1 velocity pulsatility, pulse pressure with BG, CSO and M1 velocity pulsatility, pulse wave velocity with BG and CSO damping indices, and a mean‐split scatterplot of CSO pulsatility and M1 pulsatility, and the association with pulse wave velocity. [file JMRI-55-1785-s001.docx]

Supplementary Material

| 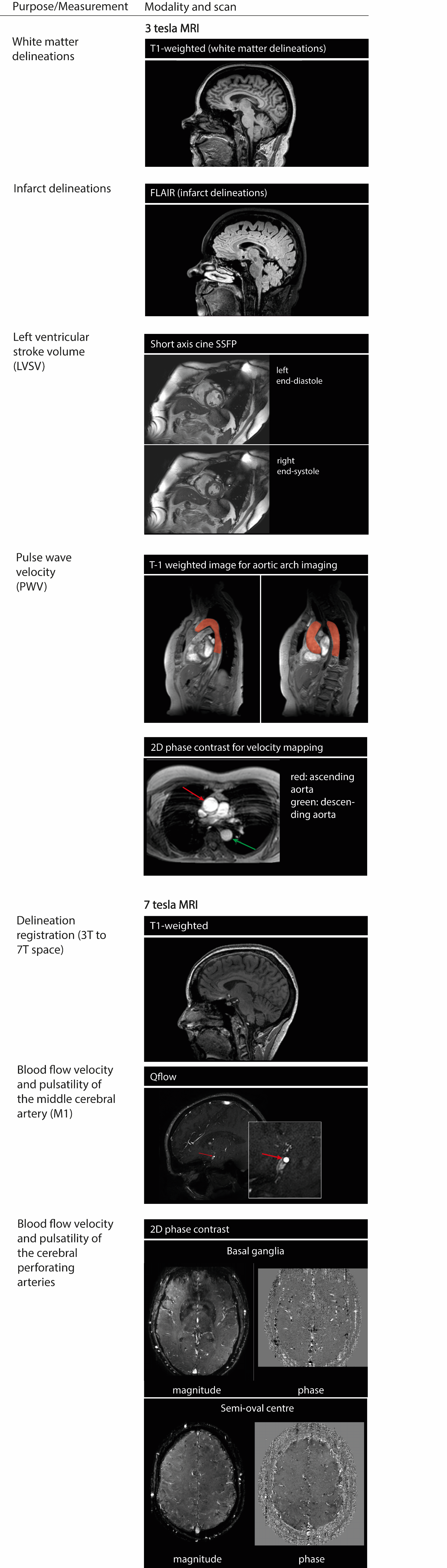 |
| --- |
| 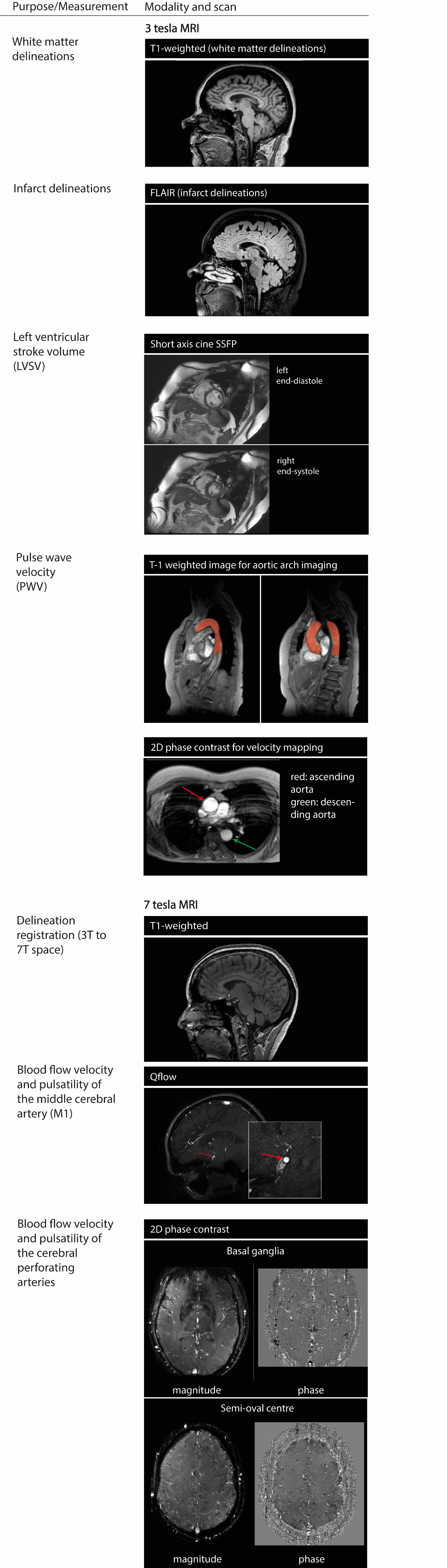 |
| 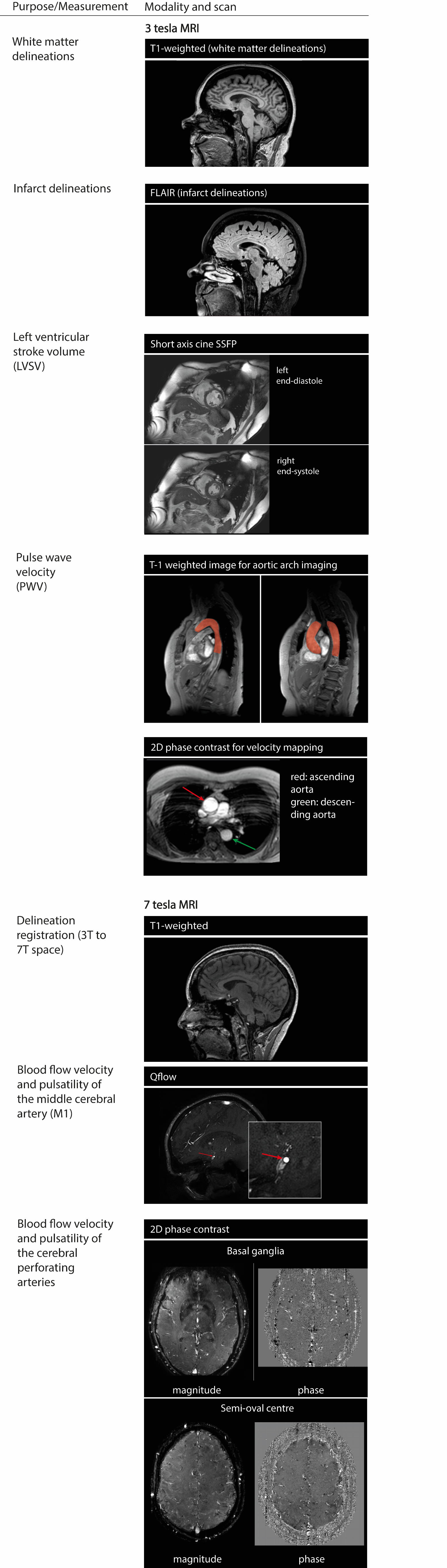 |
| Figure S1: Overview of the MRI scans used for the discussed research, on 3 tesla and 7 tesla MRI. For the scans pertaining to the middle cerebral artery, the arrows indicate the M1. |

| 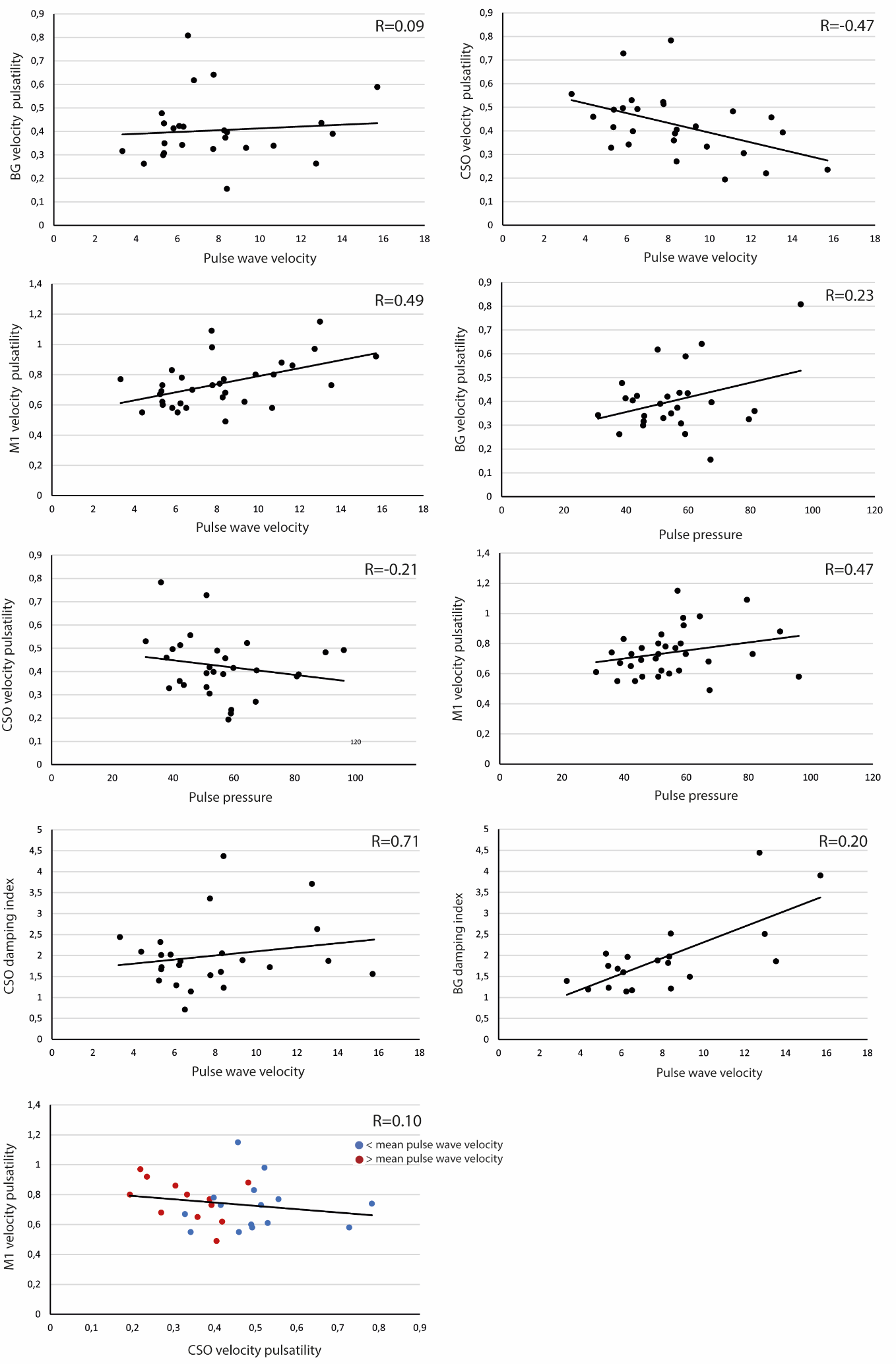 |
| --- |
| Figure S2: Scatterplots showing relations of aortic pulse wave velocity with BG, CSO and M1 velocity pulsatility, pulse pressure with BG, CSO and M1 velocity pulsatility, pulse wave velocity with BG and CSO damping indices, and a mean-split scatterplot of CSO pulsatility and M1 pulsatility and the association with pulse wave velocity. |
